# Applications of artificial intelligence and machine learning models in the prognosis and diagnosis of ovarian cancer

**DOI:** 10.3389/fonc.2026.1743601

**Published:** 2026-05-22

**Authors:** Dina M. Khodeer, Celestin Ukozehasi, Ali M. Alaseem, Sally M. Abdelmonem

**Affiliations:** 1 Department of Pharmacology, College of Medicine, Imam Mohammad Ibn Saud Islamic University (IMSIU), Riyadh, Saudi Arabia; 2 Department of Science, School of Agriculture and Food Sciences, University of Rwanda, Kigali, Rwanda; 3 Medical Physiology Department, Faculty of Medicine, Suez Canal University, Ismailia, Egypt

**Keywords:** Artificial intelligence, biomarkers, deep learning, ovarian cancer, radiomics

## Abstract

Ovarian cancer (OC) is a predominant cause of fatality amongst gynecological malignancies, frequently identified at its later stages owing to its asymptomatic characteristics and the absence of adequate screening techniques. Imaging techniques such as ultrasound (US), magnetic resonance imaging (MRI), and computed tomography (CT) are crucial for diagnosis, but traditional methods rely heavily on subjective evaluations by radiologists. AI and radiomics offer a data-driven approach to extract quantitative features from medical images, enabling more accurate and personalized diagnosis and prognosis. This review highlights the role of AI in improving the analysis of biomarkers like CA-125, HE4, and microRNAs, and discusses the potential of integrating multiomics data (genomics, transcriptomics, epigenomics, etc.) with imaging data to enhance predictive models. Radiomics, which involves extracting high-dimensional features from medical images, has shown promise in differentiating between benign and malignant tumors, predicting genetic mutations (e.g., BRCA), and assessing tumor heterogeneity. Artificial intelligence (AI) models, particularly deep learning (DL) algorithms, have demonstrated high accuracy in diagnosing OC and predicting patient outcomes, often outperforming traditional methods.

## Introduction

Ovarian cancer (OC) ranks as one of the leading causes of mortality, being the seventh most frequently diagnosed cancer in women (1). The patient’s five-year survival rate ranges from 26 to 42 percent post-diagnosis in advanced stages (2). The majority of OC cases are asymptomatic, so most cases are diagnosed at an advanced stage, which contributes to the low survival rate. The symptoms of OC include stomach bloating, early satiety, nausea, abdominal distension, changes in bowel habits, urinary issues, back pain, fatigue, and weight loss, all of which are unspecific. Notwithstanding all these symptoms, they manifest just months prior to the patient’s diagnosis in the advanced stage. An important factor for the late-stage diagnosis of OC in most patients is the lack of an efficient screening test for disease detection (3).

Radiology functions as a crucial tool in medical research, employed in clinical settings to improve taking decisions related to diagnosis, staging, and therapy. Ultrasound (US) is commonly utilized to detect masses in the ovaries and to distinguish among benign and malignant cancers (4). Magnetic resonance imaging (MRI) plays a critical role in characterizing OCs due to its superior soft-tissue resolution and is routinely recommended for determining the surgical necessity of adnexal masses (5). Computed tomography (CT), on the other hand, is particularly useful for evaluating the extensive dissemination of OC via hematogenous, peritoneal, and lymphatic routes, owing to its capacity to visualize key regions such as the liver, paraaortic lymph nodes, omentum, and mesentery (6). At present, ultrasound (US) and MRI are the main imaging modalities utilized for diagnosing and characterizing OC (7). Historically, the diagnosis of OC has relied heavily on the subjective judgment of radiologists and gynecologists, who interpret imaging features based on clinical experience to evaluate the highly heterogeneous presentation of ovarian tumors (8).

The advancements in artificial intelligence (AI) may facilitate the reconciliation of the high demand for diagnostic imaging with the comparatively constrained healthcare resources. Radiomics, an intriguing research focal point, is characterized as a novel ‘data-driven’ methodology for deriving extensive quantitative signals from radiological pictures. Following acquisition, the data can be evaluated using traditional biostatistical methods or AI approaches (9–11). Sophisticated image processing tools convert medical images into large-scale quantitative features, enabling correlations with pathological findings or treatment outcomes. Radiomics frameworks and AI-based models have demonstrated significant potential in leveraging medical imaging for OC screening (12–14).

In the present review, we aimed to elaborate on the advancements of using AI and ML in prognosis and diagnosis of OC. This review was conducted using a structured literature search to ensure comprehensive identification of relevant studies. Electronic databases including PubMed, Scopus, and Web of Science were systematically searched for articles published between January 2010 and September 2025. The search strategy combined terms related to OC with AI methodologies, including machine learning, deep learning, radiomics, radiogenomics, and multiomics. Studies were included if they evaluated AI-based models for diagnosis, prognosis, or treatment prediction in OC and reported quantitative performance metrics. The study selection process included screening of titles and abstracts followed by full-text evaluation. Data extraction included study design, sample size, AI model type, validation strategy, and performance metrics. Although this review is not a formal systematic review, efforts were made to ensure structured and reproducible study selection.

## Artificial intelligence in the diagnosis of ovarian cancer

Medical imaging is essential for the diagnosis of OC. AI and machine learning (ML) techniques, especially deep learning (DL) models, have been utilized to interpret imaging data from US, CT, and MRI. Biomarkers include CA-125, HE4, and microRNAs (miRNAs) have been utilized in the diagnosis and surveillance of OC (15, 16). AI and ML can improve the analysis of these biomarkers by detecting patterns and correlations that are not evident with conventional statistical techniques. Despite the accumulation of substantial clinical, genetic, and imaging data on OC globally, along with the advancement of high-throughput computers, such data often stay isolated and thus inaccessible for integrated studies (17).

## Image-based diagnosis using artificial intelligence and radiomics

Radiomics is a novel term in radiology, originating from the fusion of “radio,” signifying medical imaging, and “omics,” which refers to diverse disciplines such as genetics and proteomics that enhance our comprehension of various medical disorders (18).

AI encompasses a collection of systems capable of making precise inferences from extensive datasets, utilizing sophisticated computer techniques (19). Similar to humans, learning is an essential requirement for any intelligent machine behavior. Thus, AI is a broad notion that includes various learning methods, specifically ML and the increasingly popular DL algorithms (20, 21).

The relationship between radiomics and AI is reciprocal. The discipline of radiomics, characterized by its increasingly high-dimensional complexity, necessitates more robust analytical tools, and AI emerges as a promising candidate due to its exceptional capabilities. In the field of medical imaging, AI applications require radiomics, as the metrics used to train and improve AI models originate from radiomic approaches, specifically extraction of features and feature engineering methods (9, 22, 23).

The primary rationale for employing AI in radiomics is its superior capacity to manage extensive datasets in comparison to conventional statistical techniques. AI algorithms are primarily employed for classification tasks. These algorithms learn from the provided data by evaluating patterns and subsequently make predictions on unseen datasets to verify the accuracy of these patterns. AI algorithms can examine both the numeric data from established radiomic features and the images themselves to autonomously generate their own radiomic features (24–26). DL algorithms can autonomously execute segmentation tasks without human assistance (27).

### Image capture

Radiomics can be utilized across multiple imaging modalities, including CT, MRI, PET, X-ray, and US. The significance of various acquisition and image processing techniques is often undervalued or overlooked in visual analysis; however, these techniques can substantially influence radiomics, as they operate at the pixel or voxel level, potentially altering image noise and consequently texture, which may indicate differing underlying pathologies. These discrepancies may result in contradictory outcomes in radiomic analyses across independent datasets, which is a significant challenge in the field of radiomics (28, 29).

### Data Preprocessing

Radiomics is contingent upon certain picture properties. The primary factors to address in any imaging modality are the dimensions of the pixels or voxels, the quantity of gray levels, and the spectrum of gray level values. Furthermore, signal intensity nonuniformity must be eliminated in MRI. There are various approaches for managing these dependencies. The ±3sigma normalization is the most commonly employed approach for normalizing gray level values (30). Pixel resampling can be accomplished by several interpolation techniques, including linear and cubic B-spline interpolation (31).

### Segmentation

Segmentation is widely regarded as the most crucial step in the radiomics workflow, as radiomic features are primarily derived from the defined regions or volumes of interest. However, this step poses significant challenges, especially in cancers with poorly defined or indistinct boundaries. While expert-performed manual segmentation remains the gold standard, it is highly labor-intensive and time-consuming. Moreover, manual methods are prone to variability both within and between readers, which can compromise the consistency and reproducibility of extracted radiomic features (32).

### Extraction of features

It is important to recognize that radiomics operates as a hypothesis-free approach, meaning it does not rely on prior assumptions about the clinical significance of features automatically extracted by specialized image analysis algorithms. Radiomic characteristics are classified into two types. The initial category comprises predetermined or manually crafted features, developed by human image processing specialists. These are also referred to as traditional features. The second category is deep features, which have recently gained prominence as certain DL algorithms autonomously build and select features for specific tasks inside their layers, eliminating the necessity for human interaction. Recent studies have indicated the superiority of deep features over traditional features (33, 34).

### Diagnosis of ovarian cancer with radiomics utilizing radiological pictures

In terms of data modalities, five studies employed multiple imaging types, predominantly various ultrasound (US) images (35–37), hematoxylin and eosin (H&E)-stained tissue sections (38), and confocal microendoscopic imaging (39). Lu et al. developed a more comprehensive model that integrated demographic data, serum biomarkers, color Doppler imaging, and morphological characteristics, which potentially improved diagnostic accuracy, achieving an AUC as high as 0.954 (37). The data pre-processing techniques differ across research but primarily encompass re-quantization, compression, and image segmentation. This section examines various feature selection techniques, including local binary patterns transformation (36), logistic regression analysis (37), and forward sequential search (39). A study developed an autonomous classification model utilizing deep convolutional neural networks (DCNN) that can directly handle picture matrices without requiring feature extraction (38). Two studies utilizing US pictures employed support vector machine (SVM) for AI model training and achieved commendable performance (35, 36), suggesting that SVM may be effective for identifying OC through US imaging data. A separate study utilizing US pictures employed multi-layer perceptron (MLP) and attained a high AUC of 0.954 (37). The combination of demographic variables, morphological features, and serum biomarkers in building AI classifiers indicates that multi-layer perceptron architectures are well-suited for handling multi-modal data. Across studies, reported performance metrics are consistently high, with area under the curve (AUC) values frequently exceeding 0.85 and in several cases surpassing 0.90; however, these results are predominantly derived from retrospective, single-center datasets with limited external validation, which may overestimate real-world performance (40–42).

### Pathological classification

Epithelial ovarian cancer (EOC) is categorized into type I and type II based on the 2014 classification criteria for female reproductive organ malignancies established by the WHO. Given the disparity in treatment and prognosis between type I and type II EOC, it is imperative to classify the pathological type following an OC diagnosis (43).

### Prognostic modeling of ovarian cancer utilizing radiomics from radiological imaging

Two studies applied AI models, leveraging radiomics and radiological imaging, to predict the prognosis of OC. These investigations assessed the AI systems’ ability to forecast recurrence rates of high-grade serous ovarian cancer (HGSOC) three years post-surgery (44, 45). Both studies employed CT imaging, with one also incorporating additional clinical patient data (44). In the single-center study, the CT images were analyzed without any prior pre-processing (45). Regarding model development, one study utilized DL network (44), while the other applied LASSO for feature selection (45). Both employed regression-based classifiers, specifically Cox regression (44) and logistic regression (45). To improve robustness and reliability, an independent validation cohort was used in both cases. The models achieved impressive area under the curve (AUC) values of 0.825 (44) and 0.8533 (45), respectively. These results highlight the promising potential of AI approaches integrating radiomics and radiological imaging for prognostic prediction in OC patients.

## Serum tumor indicators and clinical documentation (prognosis)

Previous studies have explored the application of AI for predicting prognosis in OC by leveraging serum tumor markers and clinical data. Various ML methods have been developed for tasks such as forecasting treatment response and recurrence (46–49). Additionally, one study employed electronic medical records to predict thrombotic events in patients with HGSOC (50). Most research in this area has incorporated clinical variables including patient age, FIGO stage, tumor grade, histological subtype, and CA125 levels. One investigation used laparotomy findings processed through a genetically optimized neural network to predict OC recurrence (49). Other studies have applied grid search and k-nearest neighbor (k-NN) imputation techniques to refine parameter tuning (47, 50). Among AI classifiers, artificial neural networks (ANN) were utilized in two studies focusing on disease progression (46, 49). Hwangbo et al. (47) applied a multi-layer feed-forward (MLF) network to predict platinum-based chemotherapy responses, while K-NN and support vector machine (SVM) algorithms were employed in research by Fresard et al. and Shinagare et al., respectively (48, 50). Most models were trained on datasets exceeding 100 patients, with two notable studies using ANN (46) and MLF (47) n cohorts of 668 and 710 samples. To further improve AI-driven prognostic predictions based on tumor markers and clinical records, future efforts should focus on incorporating a wider variety of algorithms and novel biomarker panels.

## Serum and clinical documentation data models (diagnosis)

Several studies have explored the use of AI for diagnosing, staging, and classifying OC based on serum tumor markers and electronic medical record data. The data typically include cancer antigens, macrophage colony-stimulating factor (M-CSF), and phospholipids. Raw data were pre-processed through logarithmic transformation followed by normalization techniques. A variety of AI algorithms were applied as classifiers: random forest (RF) was used to predict clinical stage, histological subtype, and tumor burden (51); the Bayesian change-point model (BCP) was utilized to detect early-stage OC (41); and an ANN served for binary classification to differentiate malignant from benign tumors (52, 53). MLP and Bayesian multi-layer perceptron (BMLP) are employed for the identification of malignant tumors, with MLP also offering the capability to manage incomplete data (54, 55). Furthermore, several innovative models were created by integrating various conventional methodologies. Hybrid huberized SVM (HH-SVM), which incorporates the huberized hinge loss function and the elastic-net penalty, is capable of executing classification and feature selection concurrently (56). Two research focused on enhancing the diagnosis rate of early OC developed an artificial neural network classifier based on a multilayer perceptron architecture (57, 58). This model consists of three separately trained sub-networks capable of processing various serum indicators, yielding favorable results, with an AUC of 0.89 and 0.9449 (41). This outcome suggests the prospective utilization of AI models in multifaceted scenarios. The AI model utilizing blood tumor markers demonstrates significant potential and substantial therapeutic utility in the diagnosis of OC. **Table 1** summarizes the applications of AI in OC diagnosis and prognosis.

**Table 1:** Summary of AI applications in ovarian cancer diagnosis and prognosis, including study-level methodological characteristics such as sample size, study design, validation strategy, and model performance.

Study (author, year)	Application	Sample size (n)	Study design	Validation Strategy	AI model	AUC/performance	Key findings
37	Diagnosis	Not reported	Retrospective	Internal	MLP	AUC 0.954	Multimodal data improved diagnostic accuracy
38	Diagnosis	Not reported	Retrospective	Internal	DCNN	>90% accuracy	Deep learning enables direct image classification
35	Diagnosis	Not reported	Retrospective	Internal	SVM	>80% sensitivity	Effective classification using ultrasound imaging
36	Diagnosis	Not reported	Retrospective	Internal	SVM	>80% specificity	Texture-based classification improves performance
44	Prognosis	Not reported	Retrospective	External	DL + Cox	AUC 0.825	Predicts recurrence using CT imaging
45	Prognosis	Not reported	Retrospective	External	LASSO + Logistic	AUC 0.853	Robust recurrence prediction model
51	Diagnosis	>100	Retrospective	Internal	Random Forest	AUC >0.9	Serum biomarkers enable staging prediction
41	Diagnosis	Not reported	Retrospective	Internal	BCP	AUC 0.89–0.94	Effective early-stage detection

CT, computed tomography; MRI, magnetic resonance imaging; AUC, area under curve; OC, ovarian cancer; PET, positron emission tomography; US, ultrasound; AI, artificial intelligence; DL, deep learning; SVM, support vector machine; MLP, multi-layer perceptron; CNN, convolutional neural networks; DCNN, deep convolutional neural networks; RF, random forest; ANN, artificial neural network; HH-SVM, Hybrid huberized SVM; BCP, Bayesian change-point model; O-RADS, Ovarian-Adnexal Reporting and Data System; BRCA, breast cancer susceptibility gene; EOC, epithelial ovarian cancer.

Notably, most studies summarized in **Table 1** are based on retrospective single-center cohorts with limited external validation, which may inflate reported performance metrics. **Tables 1**, **2** have been expanded to include study-level methodological characteristics, including sample size, study design, and validation strategy, to improve transparency and enable critical comparison across studies.

**Table 2:** “Summary of multiomics-based AI models in ovarian cancer, highlighting integrated data types, validation approaches, and predictive performance across studies.

Study (author, year)	Data types integrated	Sample size	Integration strategy	Validation	Performance	Key findings
59	Radiomics + Clinical	Not reported	Radiomics-clinical fusion	External	AUC 0.74	Improved BRCA prediction
60	Radiomics + Clinical	Not reported	Nomogram	External	AUC 0.81	Outperformed single models
61	Radiomics + Clinical	Not reported	Integrated model	External	AUC 0.77	Better than radiomics alone
62	Imaging + Histopathology + Genomics	Not reported	Multimodal fusion	External	AUC 0.91	Improved survival prediction
63	Imaging + Transcriptomics	Not reported	Radiogenomics	Internal	Not reported	Enhanced subtype classification
64	Radiomics + Genomics + Clinical	Not reported	Integrated model	External	Not reported	Improved treatment prediction
65	Multiomics	Not reported	Deep learning fusion	Internal	C-index 0.57	Better than single-omics
42	Histopathology + Genomics	Not reported	Integrated model	External	AUC 0.91	Significant performance gain

CT, computed tomography; MRI, magnetic resonance imaging; AUC, area under curve; OC, ovarian cancer; N/A, not applicable; BRCA, breast cancer susceptibility gene; EOC, epithelial ovarian cancer.

## Integration of artificial intelligence with multiomics biomarkers

### Imaging and additional omics

A growing body of research has explored the integration of imaging data with a variety of complementary data types (8, 44, 59, 60, 62–64, 66). Several investigations have combined imaging with clinical parameters and serum biomarkers (67–70); two studies incorporated imaging alongside clinical information and transcriptomic data (RNA-based gene expression) (63, 71); while another two integrated imaging with clinical data, serum biomarkers, and histopathological findings (60, 62). Additionally, two studies brought together imaging data with clinical variables, serum biomarkers, and genomic information, including DNA and circulating tumor DNA (64, 72). Among imaging techniques, CT was frequently utilized (61, 73, 74) followed closely by MRI (67, 68, 70). The fusion of clinical features with radiomics has notably improved the prediction of BRCA mutations, achieving an AUC of 0.74 compared to 0.62 on training data and 0.59 on test data when using radiomics alone (59). The most favorable performance, an AUC of 0.81, was reported in Wang et al. (60), through a combined radiomics-clinical nomogram, outperforming models based solely on radiomics or clinical data. The study by Crispin-Ortuzar et al. (61) found a superior AUC measure of 0.77 for the combined model, in contrast to 0.72 for radiomics alone and 0.69 for clinical data alone. Furthermore, the integration of radiomics signatures with various data types has demonstrated potential, such as in radiomics-histopathological models (62), radiomics-clinical-genomic models (71), radiogenomic models (63), radiomics-clinicopathological-genomic models (64), and radiomics-clinical-radiological characteristics models (67), which exhibited superior performance relative to the exclusive use of radiomics, clinical, genomic, and histological models. Nonetheless, there is potential to optimize the various components of radiomics pipelines to enhance the AUC of integrative multiomics predictors.

### Genomics, epigenomics, transcriptomics, and other omics

A substantial number of studies have combined genomic and epigenomic data (including DNA and DNA methylation) with transcriptomic profiles (such as RNA expression and other omics datasets) (26, 40, 42, 65, 75–80). Three investigations integrated genomics, epigenomics, and transcriptomics to distinguish between benign and malignant conditions (77), predict patient survival (65), and classify disease subtypes (76). Two additional studies incorporated these three omics layers alongside clinical data—one to predict survival outcomes (75) and the other to estimate response to neoadjuvant chemotherapy. Furthermore, one study utilized a combination of transcriptomic and clinical data to forecast response to neoadjuvant chemotherapy (78). In another analysis, researchers integrated genomics, transcriptomics, proteomics, and pathology data to model survival outcomes in ovarian cancer (42).

A prior study demonstrated that a multiomics model outperformed individual omics approaches, achieving a C-Index of 0.571 ± 0.036 (mean ± standard deviation) (65). In a separate investigation (78), combining clinical data with gene expression significantly improved predictive accuracy by more than 19% and 45% compared to models based solely on gene expression, across two independent datasets. Another study reported an AUC exceeding 0.95 by integrating clinical and genetic information (40). Certain authors (42) attained optimal accuracy with a multiomics model that integrates histopathological image features and genomes (AUC 0.91), in contrast to a model utilizing solely histopathological image features (AUC 0.70). Furthermore, a comprehensive multiomics model incorporating DNA methylation, copy number alterations, and RNA yielded a superior AUC of 0.70 compared to the AUCs of 0.53 for methylation data, 0.64 for copy number alterations, and 0.66 for RNA data individually. **Table 2** summarizes the use of multiomics in OC.

## Multimodal data integration in AI-based ovarian cancer models

AI models increasingly rely on multimodal data integration to overcome the limitations of single-data-source approaches in OC diagnosis and prognosis. Multimodal integration typically involves the combination of imaging-derived features (radiomics or DL embeddings) with clinical variables, serum biomarkers, and multiomics data, including genomics, transcriptomics, and epigenomics (81).

Three principal fusion strategies are reported in the literature. Early fusion integrates heterogeneous data at the input level by concatenating engineered features (radiomic features with clinical or genomic variables) prior to model training. Intermediate fusion extracts modality-specific representations using separate networks or pipelines and subsequently merges latent features within a unified model. Late fusion combines predictions generated independently from modality-specific models using ensemble or voting strategies (82, 83). Among these approaches, intermediate and late fusion are most frequently applied in OC studies due to differences in data dimensionality and scale between imaging and omics datasets (84–86).

## Data imbalance and augmentation strategies

A common methodological challenge in AI-based OC research is class imbalance, particularly in datasets involving early-stage disease, rare histological subtypes, or molecularly defined patient groups. Imbalanced data can lead to biased model training and inflated performance estimates if not appropriately addressed. To mitigate this issue, several studies employ data augmentation and resampling strategies. In imaging-based DL models, augmentation techniques such as rotation, flipping, scaling, cropping, and intensity variation are frequently applied to increase data diversity and improve model generalizability. For tabular and omics data, synthetic oversampling approaches, including the Synthetic Minority Over-sampling Technique (SMOTE), class-weighted loss functions, and bootstrapping methods, are commonly reported. More recently, generative adversarial networks (GANs) have been explored to generate synthetic medical images, although their application in OC remains limited (18, 87–89). Despite these approaches, inconsistent reporting of imbalance handling remains a limitation in the literature, underscoring the need for standardized evaluation frameworks and transparent methodological reporting.

## Limitations

Data remains the paramount and essential element for the education of AI systems. Utilizing contemporary information processing technologies to exploit radiology report databases may enhance report search and retrieval, thereby assisting radiologists in diagnosis. There is a necessity to advocate for the establishment of interconnected networks that identify patient data globally and facilitate large-scale AI training tailored to diverse patient demographics, geographic regions, and diseases. Furthermore, we underscore the necessity for more diversified imaging libraries for uncommon malignancies, including OC. However, much of the current literature is limited by common methodological weaknesses, including retrospective study designs, small cohort sizes, lack of prospective validation, spectrum bias, variable label quality, segmentation variability, scanner and protocol heterogeneity, risk of data leakage, and inconsistent reporting of model calibration and clinical utility. These limitations highlight the need for more rigorous and standardized study designs to improve the reliability and generalizability of AI applications in oncology.

In image-based diagnostic tasks, AI models have demonstrated performance comparable to, or exceeding, that of expert physicians. However, such evaluations often fail to account for the multidimensional information routinely considered by radiologists when interpreting complex examinations. Non-imaging patient attributes, including demographic characteristics, clinical history, and genetic or molecular data, provide critical contextual information that is not fully captured by image-only models and can substantially enhance predictive performance when appropriately integrated (90). The high performance of contemporary AI models is frequently accompanied by substantial algorithmic complexity, involving high-dimensional feature spaces and deep neural network architectures. As a result, the internal decision-making processes underlying image-based predictions are often difficult to interpret or explain, a limitation commonly referred to as the “black-box” problem (91). This lack of transparency presents a major barrier to clinical trust, regulatory approval, and routine implementation in oncological practice. Explainable artificial intelligence (XAI) has emerged as a promising solution to address these limitations by providing model interpretability alongside predictive accuracy. XAI techniques aim to elucidate the relative importance of input features, highlight salient image regions, and identify clinically meaningful variables that drive model outputs (92). Laios et al. (93) demonstrated the clinical utility of XAI by developing ensemble AI models capable of predicting outcomes following cytoreductive surgery for OC, while simultaneously revealing patient- and procedure-specific factors contributing to surgical risk. Subsequent work further extended this framework to predict surgical effort requirements using human-centered and clinically interpretable variables. Despite these advances, many radiomics-based tools and imaging biomarker models discussed in this review continue to function as black-box systems, limiting their reliability and interpretability in real-world clinical settings. This limitation is partly attributable to the relatively early stage of XAI adoption in oncology. Improving clinician understanding of AI principles and embedding explainability within model design will be essential for meaningful clinical integration. Future studies should therefore prioritize interpretable model architectures, transparent validation strategies, and clinically relevant explanatory outputs to facilitate safe and effective translation of AI-driven radiomics into routine OC (93–96).

## Gaps and future recommendations

Current evidence demonstrates that AI-based models can achieve promising performance in OC diagnosis, prognosis, and treatment response prediction, particularly when leveraging radiomics, DL, and multimodal data integration. Multiple studies report improved accuracy, sensitivity, and prognostic stratification compared with conventional clinical models, highlighting the potential of AI to enhance personalized oncological care.

Despite these advances, several critical gaps consistently emerge from the literature. First, most studies rely on retrospective, single-center datasets, with limited external validation, restricting generalizability and increasing the risk of overfitting. Second, substantial heterogeneity in data acquisition, feature extraction, and model evaluation impedes reproducibility and cross-study comparison. Third, class imbalance and small sample sizes, particularly for early-stage disease and rare histological subtypes, are frequently underreported or inadequately addressed. Fourth, the majority of high-performing models function as black-box systems, limiting interpretability, clinician trust, and regulatory acceptance. Finally, few studies assess AI models within real-world clinical workflows, and economic or implementation feasibility analyses remain scarce.

To address these gaps, future research should prioritize prospective, multicenter studies with standardized imaging and data preprocessing protocols to enhance robustness and external validity. Adoption of harmonized reporting standards, including transparent handling of class imbalance and validation strategies, is essential. Integration of explainable AI techniques should be encouraged to facilitate clinical interpretability and decision support. Furthermore, multimodal frameworks combining imaging, omics, biomarkers, and clinical data should be developed using reproducible and scalable pipelines. Finally, AI tools must be evaluated within clinical decision-support systems, including assessment of cost-effectiveness, workflow integration, and regulatory readiness, to enable meaningful translation into routine OC care.

## Implications for future research

Future research should focus on the collection of large, diverse, and standardized datasets to train and validate AI models. This includes imaging data, clinical records, and multiomics data from different patient populations and geographic regions. There is a need for more research on XAI to improve the interpretability of AI models in medical imaging. Understanding the rationale behind AI predictions is crucial for gaining the trust of clinicians and integrating AI into clinical practice. Further studies should explore the integration of multiomics data (genomics, transcriptomics, epigenomics, etc.) with imaging and clinical data to develop more robust predictive models. This could lead to more personalized treatment strategies and better patient outcomes. AI models need to be validated in real-world clinical settings to assess their practical utility and reliability. This includes testing the models on diverse patient populations and in different healthcare environments. Research should continue to develop non-invasive diagnostic tools using AI and radiomics to reduce the need for invasive procedures like biopsies, which carry risks such as needle path metastases.

## Critical review of the published literature

AI has emerged as a promising tool in OC research, with applications spanning imaging-based diagnosis, prognostic modeling, biomarker integration, and radiogenomics. Across the reviewed studies, a wide range of models have been employed, including traditional ML algorithms such as logistic regression, support vector machines, and random forests; radiomics-based pipelines combining feature extraction with statistical modeling; and DL architectures, particularly convolutional neural networks. Collectively, these approaches demonstrate strong predictive performance, with many studies reporting AUC values above 0.85; however, the majority rely on retrospective datasets with limited external validation, raising concerns regarding generalizability and potential overfitting. Notably, multimodal models integrating imaging, clinical, and biomarker data tend to outperform single-modality approaches, reflecting the multifactorial nature of OC.

However, a critical evaluation of these models highlights important methodological distinctions and limitations. Traditional ML models, while generally more interpretable, rely heavily on handcrafted feature selection and are sensitive to variations in segmentation and preprocessing. Radiomics-based approaches offer a structured framework for quantitative imaging analysis but remain vulnerable to instability in feature extraction and lack standardization across studies. In contrast, DL models enable automated feature learning and often achieve superior performance; however, they typically require large datasets and are prone to overfitting when trained on small, single-center cohorts. Their “black-box” nature also limits interpretability, a key barrier to clinical adoption.

Despite the high-performance metrics reported, the robustness of these models is frequently uncertain. Many are developed using retrospective datasets with limited sample sizes, and only a minority undergo external validation. Methodological issues such as data leakage, inadequate separation of training and testing datasets, and lack of standardized evaluation frameworks further complicate the assessment of reliability. Few studies report calibration metrics or evaluate clinical utility through decision-curve analysis, making it difficult to determine whether these models offer meaningful improvements over existing clinical tools.

From a clinical translation perspective, most AI models remain at an early stage of development. While some demonstrate performance comparable to expert sonographers or gynecologic oncologists in controlled settings, direct comparisons with structured diagnostic systems such as O-RADS or routine clinical workflows are limited. Traditional imaging-based assessment remains the reference standard, with expert ultrasound providing nuanced interpretation of lesion morphology and vascularity, and O-RADS offering a standardized risk stratification framework. In contrast, AI models often operate in isolation, and evidence of additive benefit in real-world practice is still sparse. Multimodal models integrating imaging, clinical, and biomarker data appear most promising for clinical application, as they capture the complexity of disease biology, but they are resource-intensive and pose validation challenges.

Future research should focus on developing robust, generalizable models using large, multicenter datasets and prospective study designs. Standardization of imaging protocols, feature extraction methods, and reporting guidelines is essential to improve reproducibility. Increasing emphasis on model interpretability, transparency, and assessment of clinical utility ideally through prospective trials comparing AI-assisted workflows against established clinical tools will be critical for adoption. Advances in radiogenomics and multiomics integration may further enhance performance and support precision medicine approaches in OC.

In conclusion, AI-based models show substantial potential to improve OC diagnosis and management, but current evidence is constrained by methodological heterogeneity, limited validation, and incomplete integration with real-world clinical practice. Bridging these gaps through rigorous study design, standardization, and clinically oriented evaluation will be key to translating AI advances into meaningful patient care.

A critical limitation across the reviewed literature is the limited use of external validation cohorts, with many models relying solely on internal validation strategies, thereby restricting generalizability.”.

“Future reviews should adopt standardized evidence synthesis frameworks incorporating study-level methodological variables such as validation strategy and cohort size to improve interpretability and clinical relevance.

## Conclusion

The convergence of AI, radiomics, and multiomics holds transformative potential for improving the diagnosis and prognosis of OC. DL-based AI systems have demonstrated high diagnostic and prognostic performance, frequently achieving AUC values above 0.85; however, their clinical applicability remains constrained by limited external validation, data heterogeneity, and challenges in model interpretability. When imaging data is combined with clinical, genomic, and pathological information, the predictive accuracy of these models is significantly enhanced. Nonetheless, several obstacles persist—such as the requirement for large and diverse datasets, challenges in model interpretability, and the effective incorporation of non-imaging patient data. Despite these hurdles, progress in AI and radiomics continues to pave the way for earlier detection, more personalized treatment strategies, and better prognostic outcomes for OC patients.
